# Synergistic anti-proliferative effects of combination of ABT-263 and MCL-1 selective inhibitor A-1210477 on cervical cancer cell lines

**DOI:** 10.1186/s13104-018-3302-0

**Published:** 2018-03-27

**Authors:** Benedict Shi Xiang Lian, Angeline En Hui Yek, Hemalata Shuvas, Siti Fairus Abdul Rahman, Kalaivani Muniandy, Nethia Mohana-Kumaran

**Affiliations:** 1 School of Biological Sciences, Universiti Sains Malaysia, 11800 Gelugor, Penang Malaysia; 2 Institute for Molecular Medicine (INFORMM), Universiti Sains Malaysia, 11800 Gelugor, Penang Malaysia

**Keywords:** Cervical cancer, BH3-mimetic, ABT-263, A-1210477

## Abstract

**Objective:**

There are number of studies which report that BCL-2 anti-apoptotic proteins (e.g. BCL-2, BCL-XL, and MCL-1) are highly expressed in cervical cancer tissues compared to the normal cervical epithelia. Despite these reports, targeting these proteins for cervical cancer treatment has not been explored extensively. BH3-mimetics that inhibit specific BCL-2 anti-apoptotic proteins may hold encouraging treatment outcomes for cervical cancer management. Hence, the aim of this pilot study is to investigate the sensitivity of cervical cancer cell lines to combination of two BH3-mimetics namely ABT-263 which selectively inhibits BCL-2, BCL-XL and BCL-w and A-1210477, a selective MCL-1 inhibitor.

**Results:**

We report that combination of A-1210477 and ABT-263 exhibited synergistic effects on all cervical cancer cell lines tested. Drug sensitization studies revealed that A-1210477 sensitised the cervical cancer cell lines SiHa and CaSki to ABT-263 by 11- and fivefold, respectively. Sensitization also occurred in the opposite direction whereby ABT-263 sensitised SiHa and CaSki to A-1210477 by eightfold. This report shows that combination of ABT-263 and A-1210477 could be a potential treatment strategy for cervical cancer. Extensive drug mechanistic studies and drug sensitivity studies in physiological models are necessary to unleash the prospect of this combination for cervical cancer therapy.

**Electronic supplementary material:**

The online version of this article (10.1186/s13104-018-3302-0) contains supplementary material, which is available to authorized users.

## Introduction

Cervical cancer is the third leading cause of cancer death in females in less developed nations [1]. The cure rate with existing treatment modalities is between 60 and 90% for women who are diagnosed early. Unfortunately, prognosis is poor for women who are diagnosed with advanced or recurrent cervical cancer. The 5-year survival for women with metastatic cancer is only 16.5% [2]. Thus far, cisplatin and paclitaxel remain as the most effective treatment and standard of care for cervical cancer management. Several targeted therapies were evaluated for treatment but only exhibited limited activity [1]. Bevacizumab a recombinant and humanized monoclonal antibody against vascular endothelial growth factor (VEGF) is the only targeted therapy which has demonstrated improved overall survival (3.7 months) when combined with chemotherapy in a phase III study [3].

The BCL-2 family proteins, divided into pro- and anti-apoptotic proteins are the regulators of the intrinsic apoptosis pathway [4]. The anti-apoptotic BCL-2 proteins are up-regulated in many cancers and hence have emerged as attractive targets for therapy especially with the development of BH3-mimetics namely ABT-263 which specifically targets these proteins [5, 6].

Numbers of studies have evaluated the expression of the anti-apoptotic proteins at different stages of cervical cancer using immunoblot, immunohistochemistry (IHC) and oligonucleotide microarray profiling. Using IHC, BCL-2 and tumour suppressor protein p53 was detected to co-express in biopsies of cervical lesions but not in the normal cervical epithelium [7]. Employing the same technique, expression levels of survivin (inhibitor of apoptosis), BCL-2 and KAI 1 (tumour metastases suppressor protein) were investigated in normal cervix, chronic cervicitis, cervical intraepithelial neoplasia (CIN) and cervical cancer. Expression levels of survivin and BCL-2 were higher in the cervical cancer tissues than in the normal cervix, chronic cervicitis, or CIN and vice versa for KAI 1. Collectively, high expression of survivin and BCL-2 and low expression of KAI 1 were reported to promote cervical cancer progression and metastasis [8]. Another study using IHC, demonstrated that expression of BCL-2 was the highest in the invasive squamous cell carcinoma cervical tissues compared to normal cervical epithelium and CIN tissues [9]. MCL-1 was found to be overexpressed in cervical cancer tissues compared to its normal counterpart and the overexpression was correlated with poor prognosis. In this study IHC and immunoblot yielded similar results, bolstering confidence in the findings [10]. Collectively, these studies suggest that the anti-apoptotic proteins are relevant targets for cervical cancer treatment.

ABT-263 binds with high affinity to anti-apoptotic proteins BCL-2 and BCL-XL and with lower affinity to BCL-w [5, 11]. ABT-263 has demonstrated impressive single agent activity against lymphoid malignancies and small cell lung cancer (SCLC). Phase I/II trials report that ABT-263 was either effective as a single agent [12] or in combination with other drugs in refractory chronic lymphocytic leukaemia (CLL) [13, 14]. In most solid tumours, it has become obvious that sensitivity to ABT-263 is determined by MCL-1, which the drug binds with very low affinity. Suppression of MCL-1 or induction of MCL-1 antagonist NOXA have shown to sensitize solid cancer cells to ABT-263 [15–17]. In the present study, we performed a preliminary study to investigate the sensitivity of three cervical cancer cell lines namely SiHa, CaSki, and C33A to combination of ABT-263 (Navitoclax, AbbVie Inc) [5], and MCL-1 selective inhibitor A-1210477 (AbbVie, Inc) [18] using 2-dimensional drug sensitivity assay.

## Main text

### Methods

#### Cell lines and culture

SiHa was maintained in DMEM medium supplemented with 10% (v/v) heat-inactivated foetal bovine serum (FBS), 10 U/ml penicillin and 10 μg/ml streptomycin, 1% (v/v) HEPES, 1 mM sodium pyruvate and 2 mM l-glutamine. C33A was maintained in DMEM medium supplemented with 10% (v/v) heat-inactivated FBS, 10 U/ml penicillin and 10 μg/ml streptomycin, 2 mM l-glutamine and 4.5 g/l d-Glucose, and CaSki was maintained in RPMI medium supplemented with 10% heat-inactivated FBS, 10 U/ml penicillin and 10 μg/ml streptomycin. The human foreskin fibroblasts were maintained in DMEM medium supplemented with 20% (v/v) FBS, 1 mM sodium pyruvate and 2 mM l-glutamine. All cell culture reagents were purchased from Gibco; Thermo Fisher Scientific, Waltham, MA, USA. Cells were incubated in 37 °C, 5% CO_2_. Unnecessary passaging of cell lines was avoided; experiments were conducted within two passages of reference stocks.

#### Two-dimensional (2D) drug sensitivity assay

The 2D drug sensitivity assays were performed as described [19, 20]. Cells were seeded at 2500 cells per well in 96-well plates and left to attach for 6–7 h in a humidified incubator at 37 °C, 5% CO_2_. After attachment, cells were treated to a concentration series [0–32 μM] of single agent ABT-263 and A-1210477 and as combination along the long plate axis for 72 h. Similar drug concentrations of ABT-263 and A-1210477 as previously described by [18, 21] were used in this study. Sensitization to ABT-263 by A-1210477 was assessed by testing a fixed concentration of A-1210477 to increasing concentrations and ABT-263 and vice versa for 72 h. Cell proliferation was quantified by fluorescence of SyBr Green I nucleic acid stain (Molecular Probes, Thermo Fisher Scientific, Waltham, MA, USA) employing a plate reader with 485 nm excitation and 530 nm emission filters.

#### Statistical analyses

Comparisons of IC_50_ values of the combination with either single agent A-1210477 or ABT-263 were done by two-tailed paired T test using GraphPad Prism^®^ version 7 software. The values of p-values are indicated as ***p ≤ 0.001 or ****p ≤ 0.0001.

#### Drug interaction analyses

Synergistic drug interaction was determined using the median effect principle as previously described by Chou and Talalay [22, 23]. The CompuSyn 1.0 software (ComboSyn Inc. NJ, USA) was employed to generate Fa-CI isobologram plots.

### Results

#### Synergistic anti-proliferative effect of ABT-263 and A-1210477 in the cervical cancer cell lines

Despite number of studies reporting on high expression of the anti-apoptotic proteins in cervical cancer tissues, targeting these proteins for therapy received very little attention. Here we conducted a preliminary study to investigate the sensitivity of three cervical cancer cell lines to BH3-mimetic ABT-263 which selectively inhibits BCL-2, BCL-XL and BCL-w and A-1210477, a selective MCL-1 inhibitor. The cells were tested to single agent activity of ABT-263 and A-1210477 and as combination. In order to determine that the drugs did have an effect on cell proliferation of normal cells, the drugs were tested on human foreskin fibroblasts (HFFs).

**Fig. 1:**
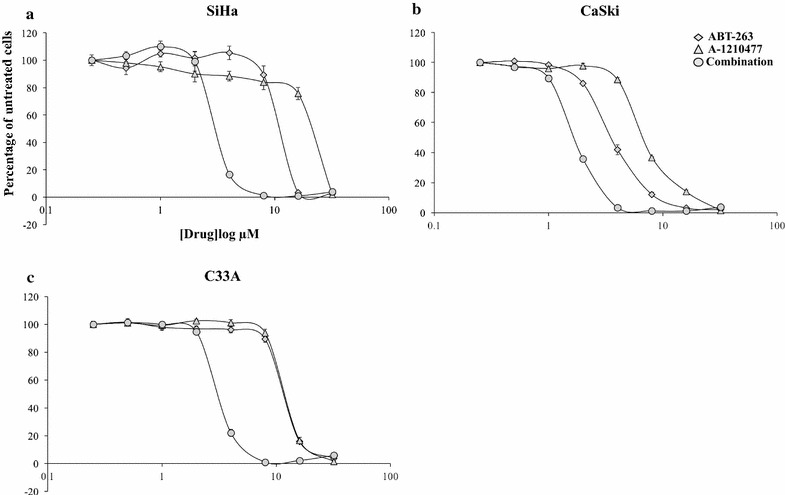
Synergistic anti-proliferative effects of ABT-263 and A-1210477 in the cervical cancer cells at 1:1 drug concentration ratios. **a** SiHa **b** CaSki and **c** C33A cell line was treated with increasing concentrations of ABT-263 (0–32 µM) (diamond) or A-1210477 (0–32 µM) (triangle) or combination of ABT-263 and A-1210477 (circle) at 1:1 drug concentration ratios for 72 h. Cell proliferation was assessed using the SyBr Green I assay. Points represent ± SEM of four repeats

All three cervical cancer cell lines were resistant to single agent activity of ABT-263 (SiHa IC_50_ 11.13 ± 0.48 µM; CaSki IC_50_ 3.4 ± 0.13 µM; C33A IC_50_ 11.77 ± 0.11 µM—Fig. 1a–c—closed diamond) and A-1210477 ((SiHa IC_50_ 20.23 ± 0.55 µM; CaSki IC_50_ 6.7 ± 0.04 µM; C33A IC_50_ 11.97 ± 0.22 µM—Fig. 1a–c—closed triangle). Combination of ABT-263 and A-1210477 at 1:1 drug concentration ratio shifted the dose–response curve to the left exhibiting selective synergistic anti-proliferative effects in all three cell lines (SiHa IC_50_ 3.07 ± 0.11 µM; CaSki IC_50_ 1.6 ± 0.04 µM; C33A IC_50_ 3.11 ± 0.06 µM—Fig. 1a–c—closed circle). The IC_50_ of the drug combination in the HFFs was 8.1 ± 0.6 µM—Additional file 1—closed circle). The IC_50_ concentration obtained for the combination in the HFFs were higher compared to the cervical cancer cell lines (Fig. 1a–c—closed circle) indicating that inhibition of cell proliferation in cervical cancer cell lines with combination of ABT-263 and A-1210477 could be achieved at lower drug concentrations without employing doses that could harm non-cancerous cells.

#### Sensitization of cervical cancer cell lines SiHa and CaSki to ABT-263 by A-1210477 and vice versa

**Fig. 2:**
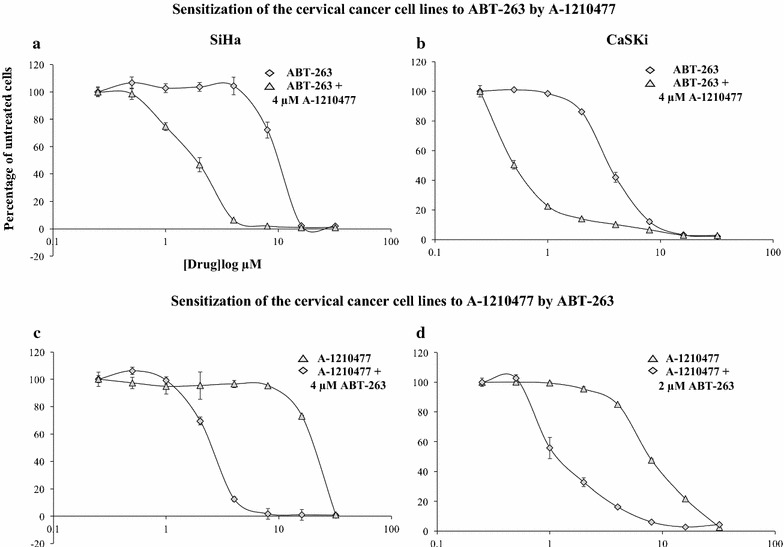
Sensitization of the cervical cancer cell lines to ABT-263 by A-1210477. **a** SiHa and **b** CaSki cells were treated with increasing concentrations of ABT-263 (0–32 µM) in the presence or absence of 4 µM of A-1210477 for 72 h. Sensitization of the cervical cancer cell lines to A-1210477 by ABT-263. **c** SiHa and **d** CaSki cells were treated with increasing concentrations of A-1210477 (0–32 µM) in the presence or absence of 4 µM and 2 µM of ABT-263, respectively for 72 h. Cell proliferation was assessed using the SyBr Green I assay. Points represent ± SEM of four repeats

Next, we investigated the sensitization effect of one drug by another in the SiHa and CaSki cells. We first tested the sensitization effect of ABT-263 by A-1210477 by adding a fix concentration of 4 μM of A-1210477 to increasing doses of ABT-263. SiHa and CaSki were resistant to single agent activity of ABT-263 (SiHa IC_50_ 10.12 ± 0.34 µM; CaSki IC_50_ 3.4 ± 0.13 µM – Fig. 2a, b—closed diamond). The presence of A-1210477 shifted the dose–response curve to the left, displaying the ability of A-1210477 to potentiate SiHa and CaSki to ABT-263 (SiHa IC_50_ 2 ± 0.15 µM; CaSki IC_50_ 0.3 ± 0.0013 µM—Fig. 2a, b—closed triangle) by 5- and 11-fold, respectively (Table 1). Sensitization was also studied in the opposite direction by adding a fix dose of ABT-263 (4 μM ABT-263 for SiHa and 2 μM ABT-263 for CaSki) to escalating doses of A-1210477. Both cells were resistant to single agent activity of A-1210477 (SiHa IC_50_ 19.86 ± 0.53 µM; CaSki IC_50_ 7.6 ± 0.11 µM—Fig. 2c, d—closed triangle). Combination with ABT-263 sensitized both cell lines to A-1210477 (SiHa IC_50_: 2.58 ± 0.2 µM; CaSki IC_50_ 0.9 ± 0.13 µM—Fig. 2c, d—closed diamond) by eightfold (Table 1). Combination index (CI) values for all combinations tested are shown in Table 1. Drug combination was synergistic at CI value of less than 1 (Additional file 2).

**Table 1:** Sensitization of cervical cell lines SiHa and CaSki to ABT-263 by A-1210477 and vice versa

	SiHa		CaSki	
*A*-*1210477*, μM	***ABT-263*** IC_50_ (μM), *fold sensitization by A*-*1210477*			
0	10.11 ± 0.34		3.4 ± 0.13	
4	2 ± 0.15	5.1***	0.3 ± 0.0013	11.3***
*ABT*-*263*, μM	***A-1210477*** IC_50_ (μM), *fold sensitization by ABT*-*263*			
0	19.85 ± 0.53		7.6 ± 0.11	
2			0.9 ± 0.13	8.4****
4	2.58 ± 0.2	7.7***		

The IC_50_ values are doses of drug 1 (bolditalics) that kill 50% of the cells surviving the shown doses of drug 2 (italics). Fold sensitization IC_50_ drug 1/IC_50_ drug 2. Errors are SEM, n = 4

Statistically significant differences with the IC_50_ of drug 1 (bolditalics) are shown as *** p ≤ 0.001 or **** p ≤ 0.0001 determined by two-tailed paired T test. Where the IC_50_ was not calculable, the lower bound was employed

Taken together our data demonstrated that the drug combination synergistic anti-proliferative effects could be explained by the ability of both drugs to sensitize each other. Hence, ABT-263 and A-1210477 may be effective sensitizers at physiologically attainable doses.

### Discussion

Neutralisation of MCL-1 is required for enhanced anti-cancer efficacy of ABT-263. Hence tumours that are normally unresponsive to ABT-263 may become amenable to treatment when combined with drugs which either repress MCL-1 or induce MCL-1 antagonist NOXA [21]. In our preliminary study, we aim to investigate the sensitivity of cervical cancer cell lines to ABT-263 when combined with MCL-1 selective inhibitor A-1210477. Our findings showed that compared to single agent treatment, combination of ABT-263 and A-1210477 caused a synergistic anti-proliferative effect in all three cell lines tested. The results obtained were in accordance with other studies [17, 24–27].

In order to fully unravel the potential of combination of ABT-263 with its partner drug, we tested the sensitization of the cervical cancer cell lines to ABT-263 by A-1210477 and vice versa. In our hands, ABT-263 sensitized cervical cancer cell lines SiHa and CaSki to A-1210477 and vice versa demonstrating that both drugs can augment the activity of each other and restore the apoptotic potential in tumour cells. This was in agreement with other studies which reported that ABT-263 sensitized the effect of docetaxel in SKOV3 ovarian cancer xenograft model and erlotinib in the NCI-H1650 NSCLC xenograft model [28]. Another study reported that ABT-263 enhanced the activity of etoposide and Bortezomib in vivo [29].

A-1210477, similar to our findings, sensitized a number of cell lines from different cancer types namely BxPC-3 pancreas adenocarcinoma line, H23-lung carcinoma line, EJ-1 gastric carcinoma line and OPM-2 multiple myeloma line to ABT-263 in vitro [18]. The sensitization effect of A-1210477 was also obvious in studies which used breast cancer [30] and non-Hodgkin’s lymphoma cell lines [31]. However, A-1210477 although highly specific for MCL-1, its ability to bind to serum proteins may limit its bioavailability and this could lead to drug resistance in preclinical models and patients as sufficient amount may not reach the tumour site.

Taken together our data demonstrates that combination of ABT-263 and A-1210477 exhibited synergistic effects in all cervical cancer cell lines tested and both drugs have the ability to enhance the activity of each other at physiologically attainable concentrations. Combination of these drugs could be a potential therapy option to combat cervical cancer but further studies are necessary to fully unleash the prospect of this duo.

## Limitations

Sensitivity of the cervical cancer cell lines to combination of ABT-263 and A-1210477 were performed in the 2D cell culture model. The 2D model is high-throughput and economical but it lacks the microenvironment that tumours encounter in vivo [32, 33]. Given that A-1210477 may demonstrate poor bioavailability in vivo, future studies in three-dimensional (3D) spheroid models, in vivo models and later in clinical trials should test combination of orally bioavailable ABT-263 with next generation MCL-1 inhibitors with improved bioavailability properties [34]. Future work should also investigate the expression of the BCL-2 proteins and caspases before and after treatment so that a mechanism for induction of apoptosis in cervical cancer cell lines by the drug combination could be established.

## Additional files

**Additional file 1.** Anti-proliferative effects of ABT-263 and A-1210477 in human foreskin fibroblasts at 1:1 drug concentration ratios. This data shows the effect of the BH3 mimetic drug combination on cell viability of a non-cancerous cell line.

**Additional file 2.** Synergistic drug effects of ABT-263 and A-1210477 in the SiHa and CaSki cervical cancer cell lines. This data shows the drug combination interaction effects in two cervical cancer cell lines.

## Abbreviations

VEGF: vascular endothelial growth factor
IHC: immunohistochemistry
CIN: cervical intraepithelial neoplasia
SCLC: small cell lung cancer
CLL: chronic lymphocytic leukaemia
2D: 2-dimensional
HFF: human foreskin fibroblasts
CI: combination index
3D: 3-dimensional
