# Supplementary material for: Synergistic anti-proliferative effects of combination of ABT-263 and MCL-1 selective inhibitor A-1210477 on cervical cancer cell lines
Source: BMC Res Notes. 2018 Mar 27;11:197. doi: 10.1186/s13104-018-3302-0 (PMC5870236; doi:10.1186/s13104-018-3302-0)
Supplement: Supplementary file 1 — Additional file 1. Anti-proliferative effects of ABT-263 and A-1210477 in human foreskin fibroblasts at 1:1 drug concentration ratios. This data shows the effect of the BH3 mimetic drug combination on cell viability of a non-cancerous cell line. [file 13104_2018_3302_MOESM1_ESM.pdf]

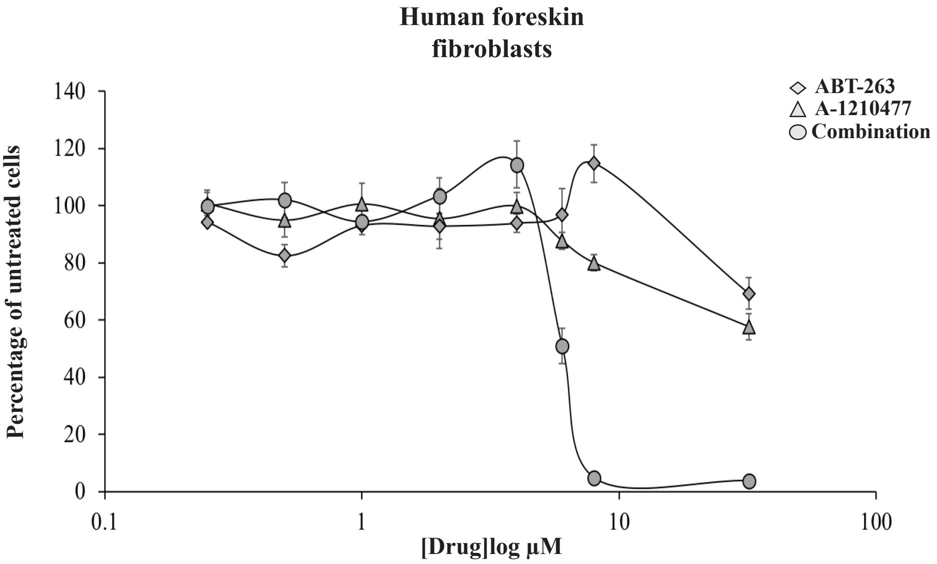

Additional file 1 Anti-proliferative effects of ABT-263 and A-1210477 in human foreskin fibroblasts at 1:1 drug concentration ratios. The cells were treated with increasing concentrations of ABT-263 (0-32 µM) (diamond) or A-1210477 (0-32 µM) (triangle) or combination of ABT-263 and A-1210477 (circle) at 1:1 drug concentration ratios for 72 hours. Cell proliferation was assessed using the SyBr Green I assay. Points represent ± SEM of four repeats.
