# Supplementary material for: Synergistic anti-proliferative effects of combination of ABT-263 and MCL-1 selective inhibitor A-1210477 on cervical cancer cell lines
Source: BMC Res Notes. 2018 Mar 27;11:197. doi: 10.1186/s13104-018-3302-0 (PMC5870236; doi:10.1186/s13104-018-3302-0)
Supplement: Supplementary file 2 — Additional file 2. Synergistic drug effects of ABT-263 and A-1210477 in the SiHa and CaSki cervical cancer cell lines. This data shows the drug combination interaction effects in two cervical cancer cell lines. [file 13104_2018_3302_MOESM2_ESM.pdf]

Additional file 2 Synergistic drug effects of ABT-263 and A-1210477 in the SiHa and CaSki cervical cancer cell lines

SiHa				CaSki			
**ABT-263 (µM)**		**CI**	**Drug combination interactions**	**ABT-263 (µM)**		**CI**	**Drug combination interactions**
0.25	A-1210477 (4 µM)	ND		0.25	A-1210477 (4 µM)	0.67	Synergism
0.5		ND		0.5		0.44	Synergism
1		1.09	Nearly additive	1		0.33	Synergism
2		0.55	Synergism	2		0.34	Synergism
4		0.27	Strong synergism	4		0.43	Synergism
8		0.33	Synergism	8		0.57	Synergism
16		0.52	Synergism	16		0.67	Synergism
32		1.03	Nearly additive	32		1.25	Moderate synergism
**A-1210477 (µM)**		**CI**	**Drug combination interactions**	**A-1210477 (µM)**		**CI**	**Drug combination interactions**
0.25	ABT-263 (4 µM)	ND		0.25	ABT-263 (2 µM)	1.29	Moderate antagonism
0.5		ND		0.5		1.50	Antagonism
1		ND		1		0.67	Synergism
2		0.95	Nearly additive	2		0.49	Synergism
4		0.35	Synergism	4		0.39	Synergism
8		0.19	Strong synergism	8		0.31	Synergism
16		0.18	Strong synergism	16		0.32	Synergism
32		0.20	Strong synergism	32		0.75	Moderate synergism

Cells were treated with increasing concentrations of ABT-263 or A-1210477 in the presence or absence of ABT-263 or A-1210477 in the indicated concentration. Combination index values were calculated using the CompuSyn 1.0 software. CI indicates combination index; CI < 1 indicates synergism, CI $= 1$ indicates additivity, and CI > 1 indicates antagonism and ND = Not determined CI values could not be determined for some combination concentrations as these concentrations were too low to cause an effect on cell proliferation.
